# Supplementary material for: Identification of Bitter Peptides in Lilium lancifolium Thunb.; Peptidomics, Computational Simulation and Cellular Functional Assays
Source: Foods. 2025 Nov 26;14(23):4056. doi: 10.3390/foods14234056 (PMC12692226; doi:10.3390/foods14234056)
Supplement: Supplementary file 1 [file foods-14-04056-s001.zip › Supplementary Tables and Figures.pdf]

Table S1. Criteria for Bitterness Evaluation.

Score	Evaluation Criteria
0~2	Absence of bitterness or off-flavor
＞2~4	Slight bitterness
＞4~6	Moderate bitterness
＞6~8	Distinct bitterness
＞8~10	Intense bitterness

Table S2. Quantitative Validation of *TAS2R14* Overexpression in Stable Cell Lines by Real-Time Quantitative Polymerase Chain Reaction (RT-qPCR) (ΔΔCt Method)

Cell Line	Ct [*TAS2R14*] (Mean ± SD)	Ct [*GAPDH*] (Mean ± SD)	ΔCt (Mean)	Relative Expression Level (Fold Change)
293T#*TAS2R14*#Pool	15.58 ± 0.15	16.17 ± 0.06	-0.59	275.45
293T#Control#Pool	23.71 ± 0.09	16.19 ± 0.08	7.52	1.00 (Reference)

Table S3. RT-qPCR primers for taste signaling genes (*TAS2R14*, *PLCβ2*, *Gustducin*, *TRPM5*).

Primer	Sequence
TAS2R14-F	TTTCATACCCTTTACTTTGTCC
TAS2R14-R	TAAGCCATTCCCATCACC
PCLβ2-F	AAGGAGATGGAGTTTCTGGAT
PCLβ2-R	ATCAGGAAAGTCCATGTTGA
Gustducin-F	CTAAAAGACTGTGGGCTTT
Gustducin-R	AAAGCAGATACTAAGATGCAC
Trpm5-F	CAAGAAGGAGGCTGAGCACAA
Trpm5-R	AGTTCTCCTTCTGGACTGTCT

Table S4. RT-qPCR primers for 7 transcriptome-based differentially expressed genes.

Primer	Sequence
ATF5-F	TGGCTCGTAGACTATGGGAAAC
ATF5-R	GCTGTGAAATCAACTCGCTCA
PPP2R2A-F	ATAAAGGTGGTAGAGTTGT
PPP2R2A-R	AGTTATACCCTTCTGGTC
CCNE2-F	AGGAGGTCACCAAGAAAC

CCNE2-R	CAGCTTAAATCAGGCAAA
PTBP2-F	CATTTGCCAAGGAGACAT
PTBP2-R	CCGAAGAGGGTAAACAGA
PPP2R5A-F	CTAACATCTTCCGTACACT
PPP2R5A-R	CTGGGATCTTCACTATCA
*PDE3*B-F	CAAGGTGGGATCGTAATA
PDE3B-R	CTTCGGGATAGTCAGTAGAT
ITPRIPL1-F	TCGGATGGACCTGGACAC
ITPRIPL1-R	CTGCCTTCTGCCTCTGCT
PDE7A-F	TTAGAAAGCAGGCAACAA
PDE7A-R	ATCGCAAAGTGGACTCAC
PDE12-F	TCGGCGATGTTAAGTACAAG
PDE12-R	GAGGAGGGAGACAATGACG
PDE6D-F	CCTGTCTGTCCCTGGTGT
PDE6D-R	GCATTGCCCTTTGAAGTA
PDE4A-F	CGTCCGTAGCAACTTCTCAC
PDE4A-R	GCTCACGGTTCAACATCCT
PDE8A-F	TGATGATGTCCCACCACG
PDE8A-R	GATTCCAAAGCGAGCAAA
PDE1B-F	TCAGCCGCTTCAAGATTC
PDE1B-R	TGTTGGTAGTGCCCGTGT
β-actin-F	ACACTGTGCCCATCTACG
β-actin-R	TGTCACGCACGATTTCC

Table S5. Total Peptide Bitterness Value Measured by Electronic Tongue.

Sample name	Electronic tongue bitterness value
LLT_E	0.35
LLT_E	0.36
LLT_E	0.29
LLT_F	4.06
LLT_F	4.01

LLT_F	4.09
LBVV_E	0.12
LBVV_E	0.13
LBVV_E	0.09
LBVV_F	2.55
LBVV_F	2.32
LBVV_F	2.41
LDVW_E	-0.55
LDVW_E	-0.46
LDVW_E	-0.43
LDVW_F	-0.35
LDVW_F	-0.36
LDVW_F	-0.33

Table S6. Bitterness scores.

Evaluators	1	2	3	4	5	6	7	8	9	10
LLT_E	8	8	8	7	9	8	8	7	8	7
LLT_F	9	10	9	8	8	9	9	9	8	10
LBVV_E	3	3	3	4	3	4	3	4	4	5
LBVV_F	5	4	5	6	5	4	5	5	4	5
LDVW_E	0	0	0	0	0	0	0	0	0	0
LDVW_F	0	0	0	0	0	0	0	0	0	0

Table S7. Bitterness scores.

Evaluators	1	2	3	4	5	6	7	8	9	10
Peptide A	4	4	4	6	4	4	5	5	5	5
Peptide B	3	4	3	4	5	4	4	4	4	3
Peptide C	4	4	5	6	4	5	4	4	5	6
Peptide D	5	6	5	5	6	4	5	5	4	5
Total Peptides	6	7	6	8	7	6	6	7	6	7
Caffeine	4	5	4	5	4	5	4	5	5	5

Figure S1. Mass spectrometry detection report of the four polypeptides（A.GAAGGSLYPNWCK. B. ENLPGGDQEKIH. C. KGTEAYLLANPDAYV. D. GGSPVWKLDSSEPNGQRYVT）.

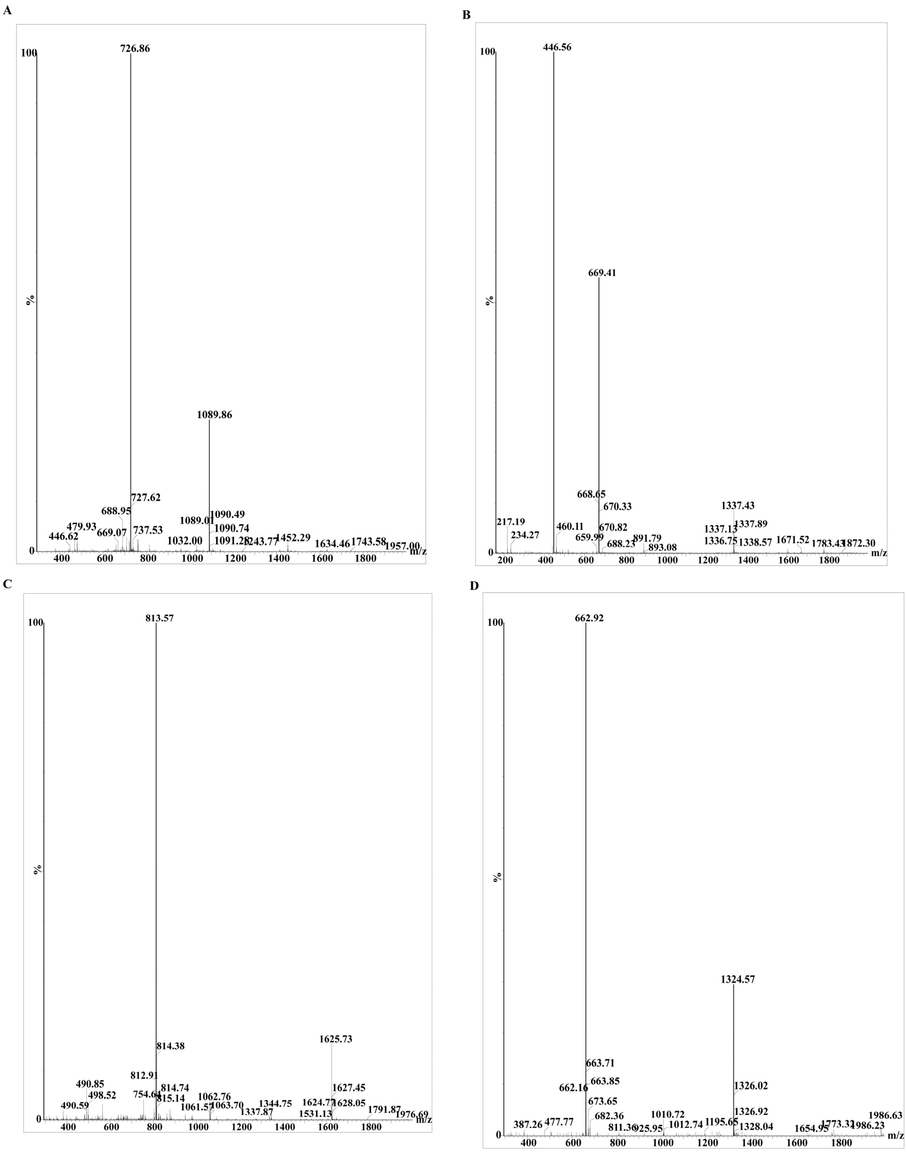

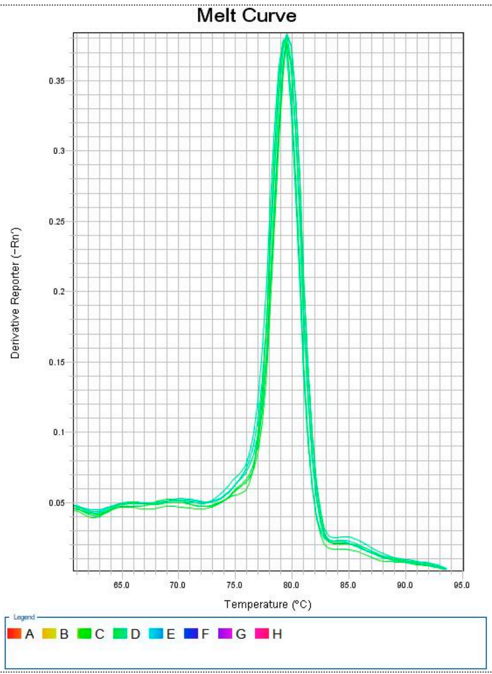

Melting Curve of hTAS2R1

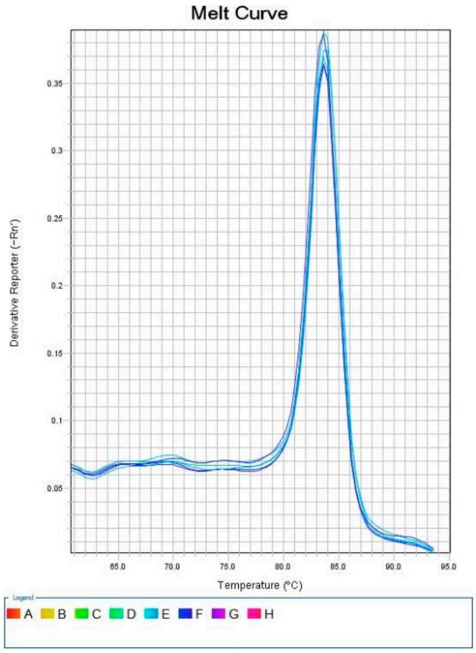

Melting Curve of hGAPDH

Figure S2. Melting Curve of TAS2R14 and GAPDH.
